# Comparing and Correcting Spectral Sensitivities between Multispectral Microscopes: A Prerequisite to Clinical Implementation

**DOI:** 10.3390/cancers15123109

**Published:** 2023-06-08

**Authors:** Margaret Eminizer, Melinda Nagy, Elizabeth L. Engle, Sigfredo Soto-Diaz, Andrew Jorquera, Jeffrey S. Roskes, Benjamin F. Green, Richard Wilton, Janis M. Taube, Alexander S. Szalay

**Affiliations:** 1 Department of Physics and Astronomy, Johns Hopkins University, Baltimore, MD 21210, USA; nagymelinda@jhu.edu (M.N.); hroskes@jhu.edu (J.S.R.); richard.wilton@jhu.edu (R.W.); szalay@jhu.edu (A.S.S.); 2 Institute for Data Intensive Engineering and Science, Johns Hopkins University, Baltimore, MD 21210, USA; 3 Department of Dermatology, Johns Hopkins University School of Medicine, Baltimore, MD 21287, USA; eengle6@jhmi.edu (E.L.E.); ssotodi1@jhmi.edu (S.S.-D.); ajorque1@jhmi.edu (A.J.); bgreen42@jhmi.edu (B.F.G.); jtaube1@jhmi.edu (J.M.T.); 4 Bloomberg-Kimmel Institute for Cancer Immunotherapy, Johns Hopkins University School of Medicine, Baltimore, MD 21287, USA; 5 Mark Foundation Center for Advanced Genomics and Imaging, Johns Hopkins University School of Medicine, Baltimore, MD 21287, USA; 6 Department of Oncology, Johns Hopkins University School of Medicine, Baltimore, MD 21287, USA; 7 Department of Pathology, Johns Hopkins University School of Medicine, Baltimore, MD 21287, USA; 8 Department of Computer Science, Johns Hopkins University, Baltimore, MD 21210, USA

**Keywords:** immunofluorescence microscopy, calibration, systematics, pathology, imaging, high throughput

## Abstract

**Simple Summary:**

Multispectral, multiplex immunofluorescence (mIF) microscopy is an emerging technology for characterization of the tumour microenvironment. Achieving high-throughput collection and analysis of mIF microscopy images often requires the use of multiple microscopes, but it is not guaranteed that data from one microscope can be compared to data from another. We used a set of eight melanoma tissue samples to measure and correct for data differences between three microscopes. We scanned the samples twice on each microscope and measured the average tissue flux densities in the resulting sets of images. By applying a relatively simple calibration model accounting for sample- and microscope-specific effects, we were able to reduce the variations in raw image brightness and immune marker expression measurements by 79% and 72%, respectively. This shows that simple procedures can be used to effectively standardize mIF data from multiple microscopes for potential use in both research and clinical diagnostic settings.

**Abstract:**

Multispectral, multiplex immunofluorescence (mIF) microscopy has been used to great effect in research to identify cellular co-expression profiles and spatial relationships within tissue, providing a myriad of diagnostic advantages. As these technologies mature, it is essential that image data from mIF microscopes is reproducible and standardizable across devices. We sought to characterize and correct differences in illumination intensity and spectral sensitivity between three multispectral microscopes. We scanned eight melanoma tissue samples twice on each microscope and calculated their average tissue region flux intensities. We found a baseline average standard deviation of 29.9% across all microscopes, scans, and samples, which was reduced to 13.9% after applying sample-specific corrections accounting for differences in the tissue shown on each slide. We used a basic calibration model to correct sample- and microscope-specific effects on overall brightness and relative brightness as a function of the image layer. We tested the generalizability of the calibration procedure and found that applying corrections to independent validation subsets of the samples reduced the variation to 2.9 ± 0.03%. Variations in the unmixed marker expressions were reduced from 15.8% to 4.4% by correcting the raw images to a single reference microscope. Our findings show that mIF microscopes can be standardized for use in clinical pathology laboratories using a relatively simple correction model.

## 1. Introduction

Multispectral, multiplex immunofluorescence (mIF) assays are emerging tools for biomarker discovery. They facilitate not only the study of basic cell population densities in a tissue-sparing manner, but also co-expression analyses, quantification of marker intensities, and spatial relationships. Multiple studies performed in numerous tumour types have demonstrated the predictive and prognostic benefit of being able to spatially resolve immunoactive cell populations within the tumour microenvironment (TME) and relate these findings to clinical outcomes [1,2,3,4,5,6,7,8,9,10,11,12,13]. In a meta-analysis of different biomarker modalities, mIF assays have been shown to have higher predictive value than tumour mutational burden, IFN-γ gene signatures, and PD-L1 immunohistochemistry for predicting response to anti-PD-1-based therapies [14].

In the research setting, performing high-throughput processing and analysis of mIF samples could lead to faster biomarker discovery. In the clinical setting, rigorously validated mIF assays could enable individualized treatments with immune checkpoint inhibitors (ICI) for patients. Given the potential for mIF assays to be used in both research and clinical settings, it is imperative to ensure these assays are reproducible.

Currently, proof-of-principle studies [15] and guidelines [16] exist around demonstrating the reproducibility of the staining portion of mIF assays. There is still an unmet need for standardizing the microscopes themselves [17,18,19]. Here, we looked to extend reproducibility assessments to the multispectral microscopes necessary for scanning the mIF-stained tissue samples. Through the use of three different microscopes housed at a single academic institution, we were able to develop a relatively simple and deployable correction model capable of adjusting these multispectral microscopes to a single reference microscope.

## 2. Materials and Methods

Eight advanced formalin-fixed paraffin-embedded (FFPE) melanoma pathology specimens were obtained from the Johns Hopkins archives. Samples were de-identified and a 4 μm section was cut from each block. Automated mIF was performed as previously described [9], but the mIF panel was expanded to include CD3 and a pan-membrane stain (Table S1).

Briefly, samples were baked offline for 3 h at 65 °C, then loaded onto the Leica BOND RX automated research stainer (Leica Biosystems, Buffalo Grove, IL, USA). Samples were then baked online at 60 °C for 30 min, and residual paraffin was removed (Dewax, Leica, Deer Park, IL, USA). Initial antigen retrieval was performed using a pH9 EDTA buffer (ER2, Leica) for 40 min at 100 °C. After initial blocking for endogenous peroxidases (BLOXALL, Vector Labs, Newark, CA, USA), non-specific antibody binding was blocked (Protein Block, Agilent, Santa Clara, CA, USA). Primary antibodies, polymers, and opals were applied for Position 1 (Table S1), then antibody stripping was performed using a pH6 sodium citrate buffer (ER1, Leica) for 20 min at 95 °C. This process was repeated for each position, after which slides were counterstained (Spectral DAPI, Akoya Biosciences, Marlborough, MA, USA) and wet mount coverslipped (Prolong Diamond, Invitrogen, Waltham, MA, USA). Prior to staining, the mIF panel was optimized to reduce cross-talk and/or bleed-through by performing primary, secondary, and fluorophore titrations to balance fluorophore intensities, as previously described [9,15]. All slides used were stained in the same batch so that batch-to-batch variations would not introduce additional artefacts.

The mIF-stained slides were scanned using PhenoImager HT (formerly known as Vectra Polaris) microscopes (Akoya Biosciences, Marlborough, MA, USA), which are automated multispectral microscopes capable of capturing fluorescent signals with wavelengths between 440 nm and 780 nm. These microscopes imaged samples by first passing light emitted from a multiband LED array through one of seven excitation filter cubes. This light illuminated areas of the tissue samples, stimulating the fluorophores and causing them to fluoresce. Fluorescence light was received by filter systems composed of seven static broadband filter cubes and 43 liquid crystal tunable narrowband filters. Light passing through each of the narrowband filters was captured by a CCD camera, forming a set of 43 monochromatic image planes. The 43-layer “raw” images were spectrally unmixed using the inForm software [20] (inForm^®^ v2.4.8, Akoya Biosciences, Marlborough, MA, USA), depending on libraries comprised of pure spectra for each fluorophore also captured on the PhenoImager HT microscopes. The spectral unmixing process transformed the raw images into 10 layers: one layer for each fluorophore, and an additional layer for autofluorescence. The 10 layers of the resulting “unmixed” images were analysed separately as measurements of individual marker expressions within the tissue samples [9,15].

Each slide was scanned twice on three different PhenoImager HT microscopes, for a total of six independent scans as summarized in Table S2. An independent scanning protocol was created for each microscope by auto-exposing on the brightest pixels for each broadband filter across the set of eight tissue samples. The broadband filters used to excite each fluorophore are listed in Table S1. Emission spectra for each fluorophore were captured across several broadband and narrowband filters, as shown in Figure S1. The microscope-dependent corrections discussed below were derived from, and applied to, the raw 43-layer multispectral images, so that a common library could be used to perform spectral unmixing on all data coming from different microscopes.

Tiling of the entire sample was achieved by acquiring 20% overlapping “high-power field” (HPF) image tiles, which were assembled into seamless whole-slide images as previously described [9]. On average, 5700 HPFs were acquired per round of scanning, totalling 34,725 HPFs across the entire dataset (Table S3). Each raw HPF image was stored as an array of unsigned 16-bit integers, with dimensions of 1872 × 1404 pixels and 43 layers. Each image layer contained the total brightness of each pixel in a specific, narrow range of light wavelengths. Image layers were grouped by the static broadband filters used to initially select wider ranges of wavelengths of light. The mIF narrow-band wavelengths contributing to each image layer and their corresponding broadband filters are plotted in Figure S1.

A binary image mask $B_{h}$ was generated for each raw HPF *h*, in which areas containing empty background or oversaturated pixels were set to 0 and areas showing well-imaged tissue were set to 1. Background pixels were determined using Otsu’s thresholding algorithm implemented in OpenCV [21]; oversaturated pixels were masked out using hand-tuned layer-dependent thresholds.

The raw HPFs were normalized by their exposure times in each image layer to produce the images $I_{h}$, with units of counts/ms. The “mean image” *M* for each scan of each sample was then calculated as

(1) $$M=\frac{\sum_{h}B_{h}I_{h}}{\sum_{h}B_{h}},$$

describing the average flux of the tissue at each pixel in counts/ms.

These mean images were averaged over the two-dimensional pixel indices *i* and *j* in each image layer *k* to produce the set of ${\bar{X}}_{mrk}^{n}$ spectra,

(2) $${\bar{X}}_{mrk}^{n}=\frac{1}{HW}\sum_{i,j}M_{mrk}^{n},$$

describing the average flux of the tissue in each image layer *k* observed for scan *r* of sample *n* on microscope *m*, where *H* and *W* are the height and width of each image.

The ${\bar{X}}_{mrk}^{n}$ spectra were then normalized so that they impacted measurements relatively equally regardless of their individual brightnesses. First, the new spectra ${\tilde{X}}_{mrk}^{n}$ were calculated by multiplying each ${\bar{X}}_{mrk}^{n}$ by the fraction of the total sample coming from its own HPFs,

(3) $${\tilde{X}}_{mrk}^{n}={\bar{X}}_{mrk}^{n}\left(\frac{h_{mr}^{n}}{\sum_{n,m,r}h_{mr}^{n}}\right),$$

and then the ${\tilde{X}}_{mrk}^{n}$ spectra were divided by their average over the N=8 samples, M=3 microscopes, R=2 scans, and K=43 raw image layers to produce the $X_{mrk}^{n}$ spectra,

(4) $$X_{mrk}^{n}=\frac{{\tilde{X}}_{mrk}^{n}}{\frac{1}{NMRK}\sum_{n,m,r,k}{\tilde{X}}_{mrk}^{n}},$$

which represent the relative tissue flux variations about one for each microscope, scan, and sample as a function of multispectral image layer.

## 3. Results

**Figure 1:**
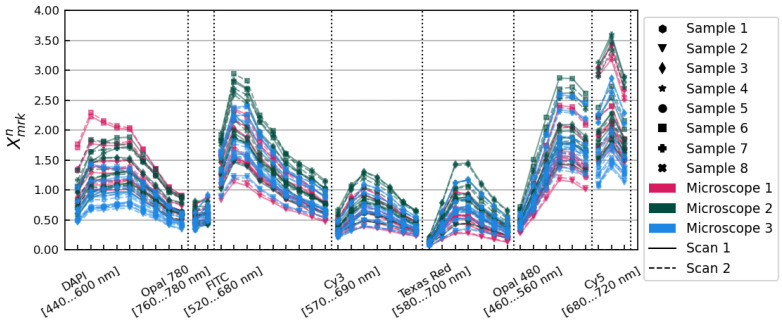
$X_{mrk}^{n}$ spectra showing average tissue flux relative to the overall mean for each scan of each sample on each microscope. Data from different microscopes are plotted in different colours. Solid and dashed lines show data from scans 1 and 2, respectively. Individual tissue samples are distinguished with different marker styles, as shown in the legend.

The $X_{mrk}^{n}$ spectra are pictured in Figure 1, showing an initial variation in overall illumination and relative spectral intensity characterized by a standard deviation of 29.85% on average over all image layers. We used these spectra to develop a method of accounting for those differences, independently modelling contributions from the individual tissue samples themselves and from the three different microscopes.

To simplify calculations, a final set of factors $a_{mr}^{n}$ were calculated, representing the normalized average relative intensities of the samples per microscope per scan without any wavelength dependence:

(5) $$a_{mr}^{n}=\frac{\frac{1}{K}\sum_{k}X_{mrk}^{n}}{\frac{1}{NMRK}\sum_{n,m,r,k}X_{mrk}^{n}}.$$

We first used a simple calibration model applied to the entire set of samples, which showed the reduction in variation that was possible to achieve overall. From this form of the calibrations, we propose a source of the observed differences between the individual multispectral microscopes. We then modified the model slightly to factor out the contributions coming only from the differences in the microscopes, and used a bootstrapping procedure to show that those microscope correction factors could be expected to generalize to additional samples. Finally, we performed three different spectral unmixings on the raw image data to evaluate how standardizing images to a single microscope affects marker intensity measurements, rather than raw image fluxes.

### 3.1. Correcting the Entire Dataset

**Figure 2:**
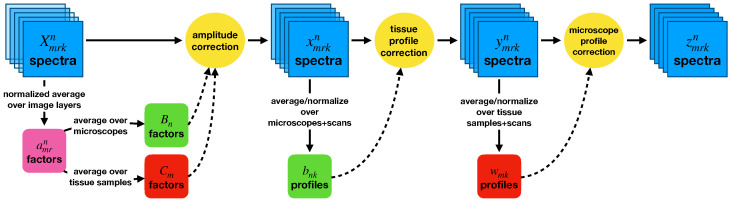
A diagram of the procedure used to correct the entire dataset. Overall amplitude corrections are applied first, then wavelength-dependent profile corrections. Contributions from the tissue samples on the slides and from the microscopes used are treated independently.

Our goal in correcting the entire dataset overall was to effect the greatest possible reduction in the variance shown in Figure 1. We first removed tissue sample- and microscope-dependent differences in the overall brightnesses of each set of images, and then accounted for differences in the relative spectral sensitivities exhibited by each tissue sample and each microscope. An overview of the method is shown in Figure 2.

We began by applying two corrections to the overall brightnesses as functions of the tissue samples, $B_{n}$, and microscopes, $C_{m}$, calculated as

(6) $$B_{n}=\frac{1}{MR}\sum_{m,r}a_{mr}^{n}\;\mathrm{and}\;C_{m}=\frac{1}{NR}\sum_{n,r}a_{mr}^{n}.$$

**Figure 3:**
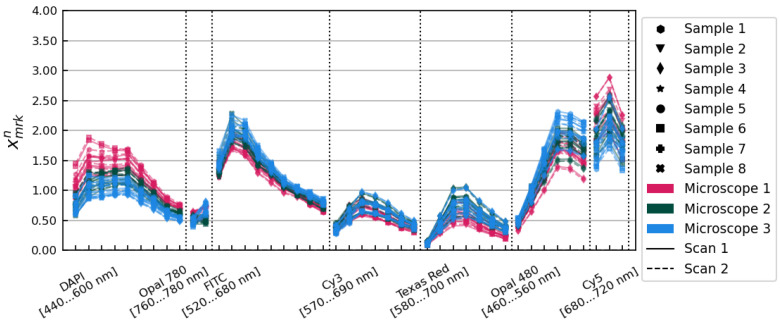
The $x_{mrk}^{n}$ spectra, after application of the $B_{n}$ and $C_{m}$ amplitude correction factors. Samples, microscopes, and scans are distinguished using the same conventions as in Figure 1.

Applying these amplitude corrections resulted in the set of $x_{mrk}^{n}=X_{mrk}^{n}/\left(B_{n}\cdot C_{m}\right)$ spectra pictured in Figure 3. These amplitude corrections alone reduced the variance observed from 29.85% to 14.83% on average over all image layers.

We next modelled the effect of varying spectral sensitivities in the specific tissues mounted on each slide. The relative variations in the sample dimension as a function of image layer, $T_{nk}$, were calculated by averaging the $x_{mrk}^{n}$ spectra over all microscopes and scans and used to determine wavelength-dependent $b_{nk}$ factors,

(7) $$b_{nk}=\frac{T_{nk}}{\frac{1}{NMR}\sum_{n,m,r}x_{mrk}^{n}}\;\mathrm{where}\;T_{nk}=\frac{1}{MR}\sum_{m,r}x_{mrk}^{n}.$$

The $T_{nk}$ variations and $b_{nk}$ correction factors are pictured in Figure S2.

**Figure 4:**
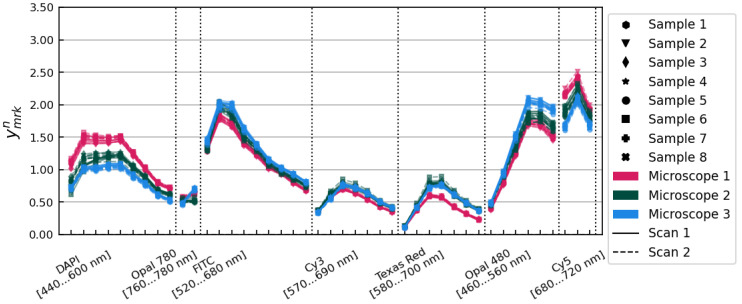
The $y_{mrk}^{n}$ spectra, after application of the $b_{nk}$ tissue profile correction factors. Samples, microscopes, and scans are distinguished using the same conventions as in Figure 1.

Applying the $b_{nk}$ tissue profile corrections to $x_{mrk}^{n}$ gave the set of $y_{mrk}^{n}=x_{mrk}^{n}/b_{nk}$ spectra, pictured in Figure 4. The standard deviation of the $y_{mrk}^{n}$ spectra was 10.56% on average over all image layers.

The variations remaining in the $y_{mrk}^{n}$ spectra corresponded to the wavelength-dependent relative differences between the three microscopes. The microscope-relative variations $P_{mk}$ and correction factors $w_{mk}$ were calculated similarly to the tissue variation spectra and corrections,

(8) $$w_{mk}=\frac{P_{mk}}{\frac{1}{NMR}\sum_{n,m,r}y_{mrk}^{n}}\;\mathrm{where}\;P_{mk}=\frac{1}{NR}\sum_{n,r}y_{mrk}^{n}.$$

The $P_{mk}$ variations and $w_{mk}$ correction factors are shown in Figure S3.

**Figure 5:**
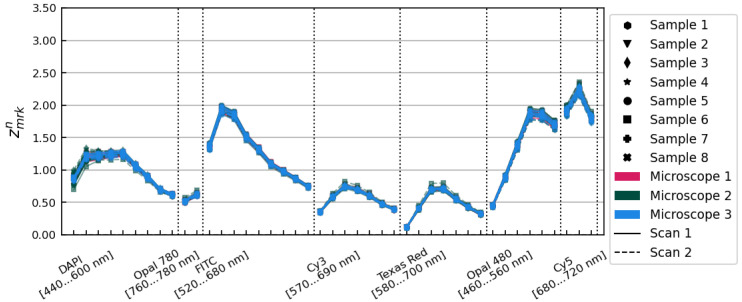
The $z_{mrk}^{n}$ spectra, after application of the $w_{mk}$ microscope profile correction factors. Samples, microscopes, and scans are distinguished using the same conventions as in Figure 1.

Applying the $w_{mk}$ factors resulted in the set of $z_{mrk}^{n}=y_{mrk}^{n}/w_{mk}$ spectra, pictured in Figure 5. The $z_{mrk}^{n}$ spectra exhibited a 2.70% standard deviation variation on average over all image layers.

**Figure 6:**
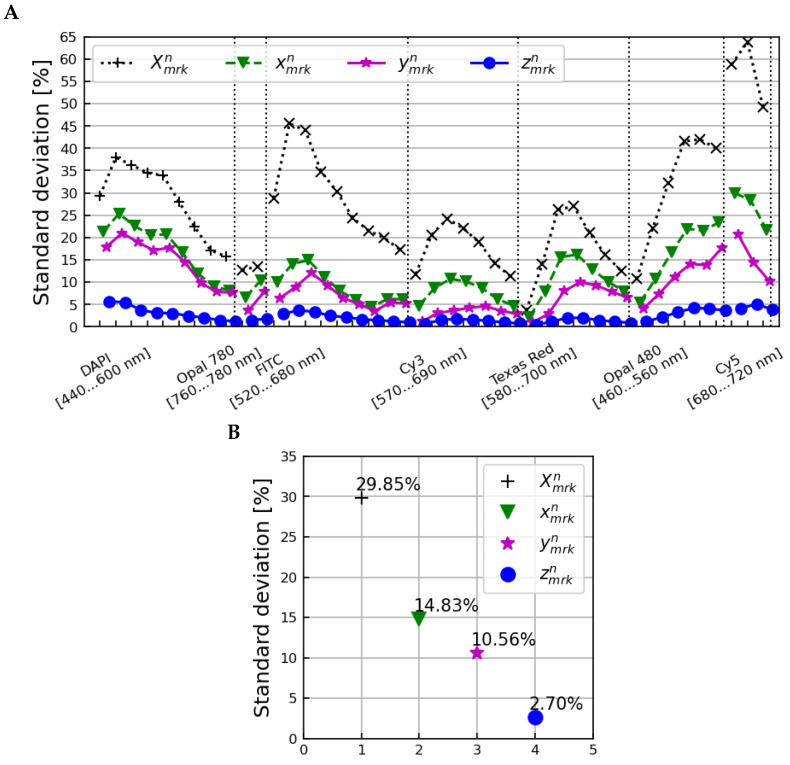
The standard deviation across all samples, microscopes, and scans as a function of image layer (**A**) and averaged over all image layers (**B**) for the $X_{mrk}^{n}$ (normalization only), $x_{mrk}^{n}$ (after amplitude corrections), $y_{mrk}^{n}$ (after correction with tissue profiles), and $z_{mrk}^{n}$ (after correction with microscope profiles) spectra.

The reduction in overall variation between all samples, microscopes, and scans is shown in Figure 6. The upper plot shows the standard deviation over all samples, microscopes, and scans as a function of the image layer at each stage of correction, and the lower plot shows the averages of these standard deviations over all image layers. An initial standard deviation of 29.85% was reduced to 2.70% after applying corrections accounting for differences between tissue samples and microscopes.

### 3.2. Contributions to Microscope-Specific Correction Factors

The $w_{mk}$ factors can be averaged over all layers to calculate $w_{m}^{\mathrm{ill}}=\frac{1}{K}\sum_{k}w_{mk}$, which should be approximately equal to 1 since the $C_{m}$ amplitude corrections were already applied in $y_{mrk}^{n}$. Dividing out these overall scales and averaging over the layers $k^{†}$ belonging to each broadband filter group produced the set of $w_{mk}^{\mathrm{BB}}$ factors,

(9) $$w_{mk}^{\mathrm{BB}}=\frac{1}{K^{†}}\sum_{k^{†}}\frac{w_{mk}}{w_{m}^{\mathrm{ill}}},$$

which quantify the differences between microscopes that are attributable to inhomogeneities in those microscopes’ specific broadband filters. Lastly, the differences specific to the piezoelectrically tuned narrow-band filters ($w_{mk}^{\mathrm{NB}}$) were quantified by dividing out both the overall illumination and broadband filter contributions:

(10) $$w_{mk}^{\mathrm{NB}}=\frac{w_{mk}}{w_{m}^{\mathrm{ill}}\cdot w_{mk}^{\mathrm{BB}}}.$$

The $w_{m}^{\mathrm{ill}}$, $w_{mk}^{\mathrm{BB}}$, and $w_{mk}^{\mathrm{NB}}$ contributions to the total $w_{mk}$ correction factors are pictured in Figure S4. It is clear that most of the differences in relative spectral sensitivities between microscopes can be attributed to inhomogeneities in the microscopes’ broadband filter cubes. Each microscope was manufactured with its own static set of broadband filter cubes, and it is expected that the materials used for those filter cubes may differ between instruments from the time of manufacture. Our data indicate that those differences can be as large as 20% with respect to the means of all three microscopes for any given broadband filter group.

This same effect is also visible when calculating the overall covariance matrices of the $X_{mrk}^{n}$, $x_{mrk}^{n}$, $y_{mrk}^{n}$ and $z_{mrk}^{n}$ spectra in the image layer dimension. The image layer-projected covariance matrix of $X_{mrk}^{n}$, for example, $\Sigma_{k_{1}k_{2}}\left(X\right)$, can be calculated as

(11) $$\Sigma_{k_{1}k_{2}}\left(X\right)=\frac{1}{NMR}\sum_{n,m,r}d_{mrk_{1}}^{n}d_{mrk_{2}}^{n},$$

where

(12) $$d_{mrk}^{n}\left(X\right)=X_{mrk}^{n}-\mu_{mk}\left(X\right)\;\mathrm{and}\;\mu_{mk}\left(X\right)=\frac{1}{NR}\sum_{n,r}X_{mrk}^{n}.$$

These image layer-projected covariance matrices at each stage of correction $\Sigma_{k_{1}k_{2}}\left(X\right)$, $\Sigma_{k_{1}k_{2}}\left(x\right)$, $\Sigma_{k_{1}k_{2}}\left(y\right)$, and $\Sigma_{k_{1}k_{2}}\left(z\right)$ are shown in Figure S5. They show an overall reduction in the scale of the variance as successive corrections are applied, as well as a strong correlation between groups of layers imaged with the same broadband filter, and between image layers corresponding to similar narrow-band wavelengths, as pictured in Figure S1.

### 3.3. Generalizing Microscope Correction Factors

**Figure 7:**
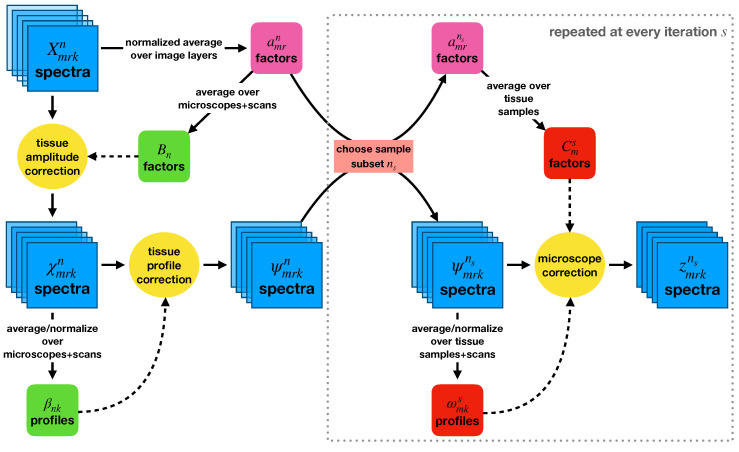
A flowchart outlining the bootstrapping method used to investigate how the microscope-specific corrections would generalize to new data. Tissue-specific normalization and profile corrections were first applied to the entire dataset. Random subsets of samples were then chosen at each bootstrapping iteration, and microscope-specific corrections were calculated using them.

Having determined a model for using a group of multiple-imaged tissue samples to measure microscope-dependent correction factors, we next investigated how generalizable the procedure would be if applied to additional data that were not used to measure the corrections. To this end, we used a bootstrapping procedure to repeatedly calculate microscope-dependent correction factors using particular subsets of the eight tissue samples and then applying those corrections to orthogonal subsets of the tissue samples. This procedure is displayed in Figure 7.

The data used in this procedure were normalized as before ($X_{mrk}^{n}$) and divided by the same tissue sample-dependent amplitude corrections $B_{n}$ to produce the set of $\chi_{mrk}^{n}$ spectra,

(13) $$\chi_{mrk}^{n}=\frac{X_{mrk}^{n}}{B_{n}}.$$

These spectra defined new $\beta_{nk}$ factors,

(14) $$\beta_{nk}=\frac{\frac{1}{MR}\sum_{m,r}\chi_{mrk}^{n}}{\frac{1}{NMR}\sum_{n,m,r}\chi_{mrk}^{n}},$$

analogous to the $b_{nk}$ factors, to account for spectral variations attributable to differences between the tissue samples on each slide. Dividing by these factors produced the set of $\psi_{mrk}^{n}$ spectra,

(15) $$\psi_{mrk}^{n}=\frac{\chi_{mrk}^{n}}{\beta_{nk}},$$

in which any remaining variations were attributable to the different microscopes.

At each iteration *s* of the bootstrapping procedure, $N^{\prime }=5$ “fit” samples $n_{s}$ were randomly chosen from the full set of eight, and the microscope-dependent correction factors $C_{m}^{s}$ and $\omega_{mk}^{s}$ were calculated using just those five samples:

(16) $$C_{m}^{s}=\frac{1}{N^{\prime }R}\sum_{n_{s},r}a_{mr}^{n_{s}}\;\mathrm{and}\;\omega_{mk}^{s}=\frac{\frac{1}{N^{\prime }R}\sum_{n_{s},r}\psi_{mrk}^{n_{s}}}{\frac{1}{N^{\prime }MR}\sum_{n_{s},m,r}\psi_{mrk}^{n_{s}}}$$

The correction factors were then applied back onto these five samples to produce the $z_{mrk}^{n_{s}}$ spectra,

(17) $$z_{mrk}^{n_{s}}=\frac{\psi_{mrk}^{n_{s}}}{C_{m}^{s}\omega_{mk}^{s}},$$

and also to the three orthogonal “test” tissue samples. The post-correction standard deviations across the fit and test samples, plus all microscopes and scans, were calculated. This procedure was repeated 56 times, once for each independent choice of the five fit samples.

**Figure 8:**
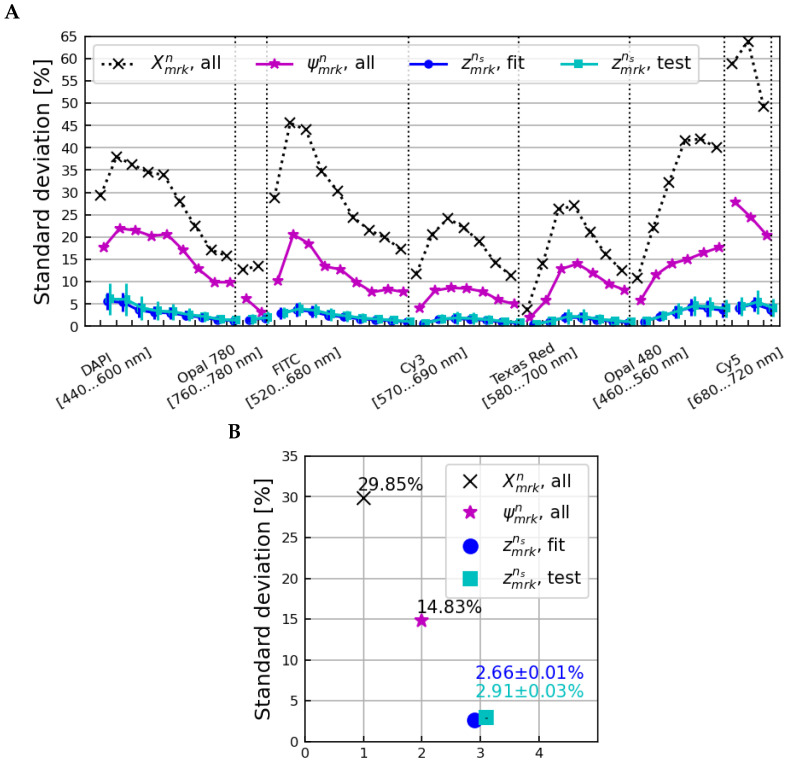
The standard deviation across all samples, microscopes, and scans as a function of the image layer (**A**) and averaged over all image layers (**B**) for the $X_{mrk}^{n}$ (normalization only), $\psi_{mrk}^{n}$ (after corrections for tissue-specific differences), and $z_{mrk}^{n_{s}},\mathrm{fit}$ and $z_{mrk}^{n_{s}},\mathrm{test}$ spectra. The $z_{mrk}^{n},\mathrm{fit}$ data points shown are the mean over all bootstrap iterations of applying the calculated corrections back onto the samples used to calculate them, whereas the $z_{mrk}^{n},\mathrm{test}$ data points correspond to corrections applied to subsets of samples orthogonal to those used to calculate the corrections at each iteration.

Figure S6 shows the distribution of the $C_{m}^{s}\omega_{mk}^{s}$ factors calculated for each iteration; the microscope-dependent correction factors were all very similar regardless of the subset of samples used to calculate them. Figure 8 shows the standard deviations across all samples, microscopes, and scans of the original $X_{mrk}^{n}$ data, the tissue-homogenized $\psi_{mrk}^{n}$ spectra, and the fully-corrected $z_{mrk}^{n_{s}}$ spectra for all fit/test sample subsets. The $z_{mrk}^{n_{s}}$ data points shown are the averages over all bootstrapping iterations, with error bars equal to the standard deviation.

Applying microscope-dependent corrections calculated using orthogonal subsets of samples reduced the standard deviation from 13.87% to 2.91 ± 0.03% on average over all image layers, comparable to the final standard deviation of 2.66 ± 0.01% observed when applying corrections back onto the subsets of samples used to calculate them. This shows that normalizing and homogenizing a set of tissue samples using $B_{n}$ and $\beta_{nk}$ correction factors reliably leaves only microscope-dependent variations present, and that corrections for those microscope variations can be reliably applied to new tissue samples from the same microscopes.

### 3.4. Impact to Measurements of Marker Expressions

Immunofluorescence microscopy is often used to measure the expressions of multiple biomarkers simultaneously. The PD1/PDL1 immunofluorescence panel used to stain the tissue samples described in Section 2 contained stains targeting CD3, PDL1, FoxP3, CD8, PD1, CD163, and Sox10/S100 proteins, as well as a DAPI stain targeting cellular nuclear DNA, and a lab-developed combination (“pan-membrane”) stain targeting cellular membranes. The inForm Automated Image Analysis Software (inForm^®^ v2.4.8, Akoya Biosciences, Marlborough, MA, USA) [20] from Akoya Biosciences was used to “unmix” the raw, 43-layer images into new, 10-layer images depicting the normalized expressions of each marker plus a layer for autofluorescence. We then quantified the effects of applying corrections for differences between microscopes on those measurements of marker expressions.

**Figure 9:**
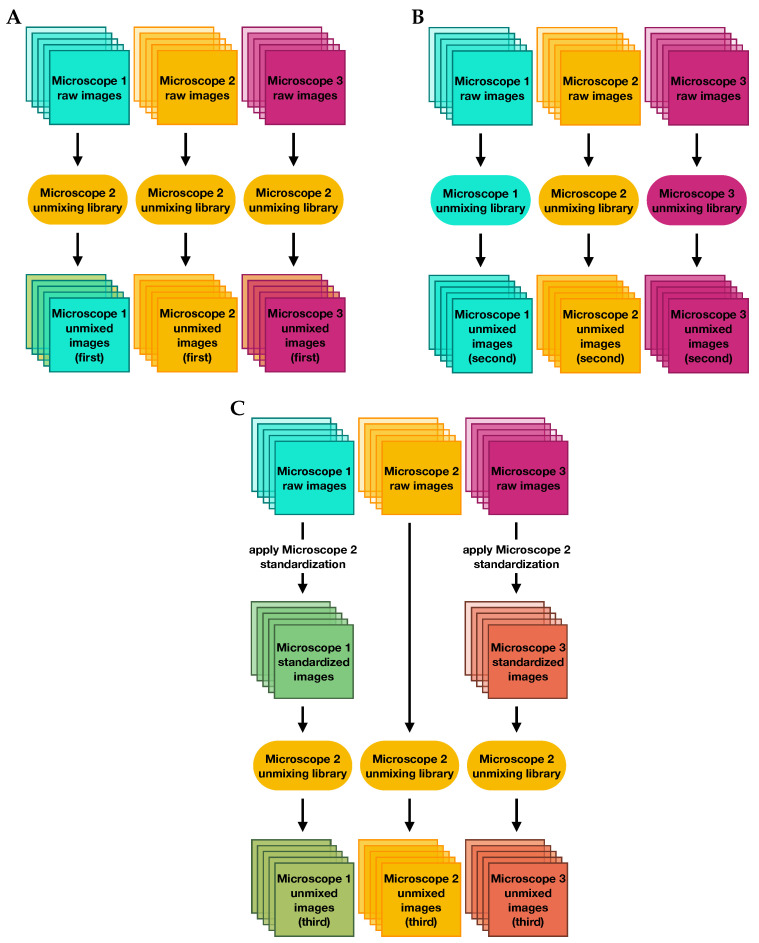
Flowcharts describing the three unmixing methods used to evaluate the impact of microscope standardization on measurements of marker expressions: (**A**) unmixing raw images with the reference microscope library, (**B**) unmixing raw images with microscope-specific libraries, and (**C**) unmixing corrected images with the reference microscope library.

The spectral unmixing process depends on “library” slides as input, which provide measurements of individual marker responses and autofluorescence at different wavelength ranges. We investigated three different unmixing scenarios, depicted in Figure 9.

First, we unmixed the raw data using a single library whose slides were imaged on microscope 2. Then we performed a second unmixing using three different libraries whose slides were imaged on each of the three microscopes, where raw data were unmixed using the library from the microscope on which they were scanned. In the final scenario, we first applied factors to standardize all images to measurements from microscope 2, and then unmixed all of the corrected images using the single library from microscope 2.

The standardization factors applied were the means of the $C_{m}^{s}$ and $\omega_{mk}^{s}$ factors shown in Figure S6, divided by the factors for microscope 2, so that microscope 2 data were left unaltered and data from microscopes 1 and 3 were standardized to that single reference. The standardization was performed by dividing each raw image by the product of the $C_{m}^{\prime }$ and $\omega_{mk}^{\prime }$ factors, as in Equation (17).

**Figure 10:**
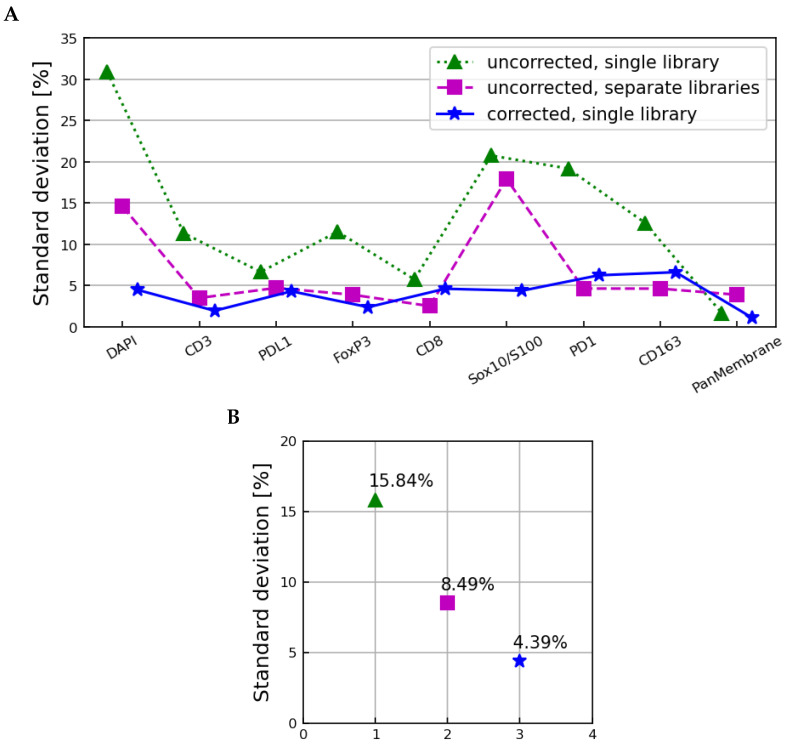
The standard deviation across all samples, microscopes, and scans as a function of the image layer (**A**) and averaged over all image layers (**B**) for the $\psi_{mrk}^{\prime n}$ spectra derived from uncorrected images unmixed with the single set of microscope 2 libraries (dotted line), uncorrected images unmixed using microscope-specific libraries (dashed line), and corrected images unmixed using the microscope 2 libraries (solid line). The autofluorescence layer is omitted in (**A**) and not used to calculate the values in (**B**).

The three sets of unmixed images were multiplied by their binary image masks and their average brightnesses in each layer were calculated and normalized as in Equations (1)–(4) above, except that the number of image layers was $K^{\prime }=10$ instead of K=43. Tissue sample-specific normalization factors $B_{n}^{\prime }$ and $\beta_{nk^{\prime }}^{\prime }$ were calculated as in Equations (6) and (14), respectively, and applied as in Equation (15). The resulting three sets of $\psi_{mrk^{\prime }}^{\prime n}$ spectra, one for each unmixing method, are shown in Figure S7 (the autofluorescence layer is omitted). The standard deviations across all samples, microscopes, and scans of these spectra are shown in Figure 10, along with their averages over all but the autofluorescence layer.

The uncorrected images unmixed with the microscope 2 library showed a remaining variation characterized by an average standard deviation of 15.84%, slightly larger than that observed in the tissue sample-corrected $y_{mrk}^{n}$ and $\psi_{mrk}^{n}$ spectra in Figure 6 and Figure 8, respectively. The uncorrected images unmixed with the individual microscope libraries had an average standard deviation of 8.49%, showing that using microscope-specific libraries in unmixing does compensate for some, but not all, systematic differences between samples imaged on different microscopes. The microscope-corrected images unmixed using the microscope 2 library showed an average standard deviation of 4.39%, slightly larger than the fully corrected $z_{mrk}^{n}$ spectra in Figure 6 and Figure 8.

The greatest reduction in the unmixed images’ microscope-specific differences was therefore observed by standardizing the fluxes of the raw images to a single reference microscope, and then unmixing all images using a library from that single reference microscope. The variations remaining in the unmixed images were slightly larger than those remaining in the raw images; likely due to the dimension reduction from 43 to 10 image layers that is inherent to the unmixing process.

## 4. Discussion

The use of immune checkpoint inhibitors (ICI), has completely changed the landscape of treatment for patients with advanced melanoma and other tumour types [22]. Two recently published clinical trials treating naïve patients with advanced melanoma showed a five-year overall survival (OS) >40% in patients treated with anti-PD-1 [23,24]. Additionally, patients treated with a combination of anti-PD-1 and anti-CTLA-4 showed an even higher median 6.5-year OS compared to patients treated with anti-PD-1 alone [25]. A pre-treatment biomarker to help predict which patients are more likely to respond to therapy is of great interest. Currently, there are no FDA-approved companion diagnostics to determine if patients with advanced melanoma should receive ICI [26]. Initially, a PD-L1 immunohistochemistry assay was approved as a complementary diagnostic, but this was ultimately rescinded after levels of PD-L1 expression did not correlate with OS [27]. More recently, a 6-plex mIF assay for predicting objective response, progression-free survival, and OS for patients with advanced melanoma receiving anti-PD-1-based ICI was developed [9].

There are many steps involved with creating a companion diagnostic assay including, but not limited to, demonstrating high intra- and inter-observer reproducibility of the assay [28,29,30]. This includes demonstrating little variability between multiple reagent lots, validating all instruments involved with performing the assay, and potentially creating a “locked-down” analysis algorithm for those assays requiring image analysis. In collaboration with several other groups, we have performed the initial steps for validating and determining the reproducibility of a mIF 6-plex assay by showing a strong inter- and intra-site concordance of both cell population densities and marker intensity measurements [15]. Some limitations of this study were that only the reproducibility of the mIF staining itself was tested, and that only regions of interest within the mIF-stained slides were scanned and analysed. Here, we expanded the scanned image to include the whole slide and standardize the multispectral microscopes used to acquire the imagery.

Through the serial scanning of eight advanced melanoma FFPE mIF-stained sections we were able to characterize systematic differences between three PhenoImager HT microscopes and showed that these differences are due to inhomogeneities in the broadband filter cubes built into each microscope. We developed a simple correction model that shows measurements of microscopes-specific correction factors are relatively agnostic to the specific samples used to measure them. Additional work may be needed to determine if these factors remain agnostic when scanning is performed on tissue from other tumour types, as there can be significant differences in staining patterns and background autofluorescence between tumour types. The proposed correction model factors out differences in tissue area across samples and differences between microscopes, making it possible to standardize image data from multiple microscopes to the mean of all microscopes or to a single reference microscope. By standardizing these data to a single reference microscope, we were able to reduce microscope-dependent flux variation in raw images by 79%, and in marker expressions measured in the spectrally unmixed images by 72%. Microscope-specific corrections of this form could allow for the harmonization of mIF assay results across institutions. With such harmonization, it may be possible to use a single set of software phenotyping projects across all samples, which is a pre-requisite for the development of “locked-down” analysis algorithms. More work will be needed to measure and test corrections of this form for microscopes housed at different institutions, and any microscope-specific standardization procedures must remain independent of other standardization steps performed to ensure reproducibility of marker panels or other aspects of imaging. Our group is also developing a method to standardize image data from slides stained in multiple batches, which will be the subject of a forthcoming publication.

These investigations imply a procedure to allow high-throughput mIF imaging using more than one PhenoImager HT microscope. For example, if a large number of slides are obtained all at once for imaging, several slides can be reserved for imaging on all available microscopes to determine microscope-dependent correction factors, and the rest can be imaged on only one microscope. HPFs from microscopes other than the chosen reference microscope can be corrected by the measured standardization factors, and then unmixed using only one library imaged on the reference microscope. The results presented here are only applicable to the specific PhenoImager HT microscopes at a single academic institution. It is expected that other PhenoImager HT systems would exhibit comparable differences due to their own static broadband filter cubes, and that the same method of measuring and applying corrections before spectral unmixing using multiple-imaged samples of a single tissue type would be a reasonable method for quantifying those differences as realized within that tissue type. Additional factors would need to be considered in developing correction models for multispectral image data from other systems.

## 5. Conclusions

We have characterized the differences in tissue fluxes observed in mIF microscopy data collected using three different multispectral microscopes at JHU. We used a basic sequential calibration model to measure and apply sample- and microscope-specific effects on the overall brightness and relative brightness as a function of image layer/narrow-band wavelength. We investigated the effects of generalizing the calibration procedure to additional data using a bootstrapping method. It was observed that an initial standard deviation in the average tissue fluxes across all microscopes, scans, and samples of 29.85% on average over all image layers was reduced to 13.87% after applying sample-specific corrections accounting for differences in the tissue shown on each slide. Applying microscope-specific corrections to orthogonal sample subsets further reduced the variation to 2.91 ± 0.03%. Variation in marker expressions observed in corresponding spectrally unmixed images was reduced from 15.8% to 4.4% by correcting raw images to a single reference microscope before unmixing. Our findings show that mIF microscopes can be standardized for use in clinical pathology laboratories using a relatively simple correction model that can reduce variation between microscopes by 79% in raw images and 72% in spectrally unmixed images.

## Data Availability

The data presented in this study are available on request from the corresponding authors.

## Supplementary Materials

The following supporting information can be downloaded at: https://www.mdpi.com/article/10.3390/cancers15123109/s1, Table S1: Antibodies and staining conditions for automated mIF assay; Table S2: Dates that each sample was scanned on the three microscopes; Table S3: Number of HPFs generated for every scan of each sample used; Figure S1: Narrow-band filter wavelengths contributing to each HPF image layer; Figure S2: $T_{nk}$ spectra and $b_{nk}$ correction factors; Figure S3: $P_{mk}$ spectra and $w_{mk}$ correction factors; Figure S4: $w_{m}^{\mathrm{ill}}$, $w_{mk}^{\mathrm{BB}}$, and $w_{mk}^{\mathrm{NB}}$ contributions to $w_{mk}$ correction factors; Figure S5: Covariance matrices at each stage of correction; Figure S6: $C_{m}^{s}\omega_{mk}^{s}$ bootstrapping correction factors; Figure S7: unmixing results.

## Abbreviations

The following abbreviations are used in this manuscript:

mIF	Multispectral/multiplex immunofluorescence
TME	Tumour microenvironment
ICI	Immune checkpoint inhibitors
FFPE	Formalin-fixed paraffin-embedded
HPF	High-power field
OS	Overall survival

## References

[1] Johnson D.B., Bordeaux J., Kim J.Y., Vaupel C., Rimm D.L., Ho T.H., Joseph R.W., Daud A.I., Conry R.M., Gaughan E.M. (2018). Quantitative Spatial Profiling of PD-1/PD-L1 Interaction and HLA-DR/IDO-1 Predicts Improved Outcomes of Anti–PD-1 Therapies in Metastatic Melanoma. Clin. Cancer Res..

[2] Giraldo N.A., Nguyen P., Engle E.L., Kaunitz G.J., Cottrell T.R., Berry S., Green B., Soni A., Cuda J.D., Stein J.E. (2018). Multidimensional, quantitative assessment of PD-1/PD-L1 expression in patients with Merkel cell carcinoma and association with response to pembrolizumab. J. Immunother. Cancer.

[3] Zheng X., Weigert A., Reu S., Guenther S., Mansouri S., Bassaly B., Gattenlöhner S., Grimminger F., Savai Pullamsetti S., Seeger W. (2020). Spatial Density and Distribution of Tumor-Associated Macrophages Predict Survival in Non–Small Cell Lung Carcinoma. Cancer Res..

[4] Althammer S., Tan T.H., Spitzmüller A., Rognoni L., Wiestler T., Herz T., Widmaier M., Rebelatto M.C., Kaplon H., Damotte D. (2019). Automated image analysis of NSCLC biopsies to predict response to anti-PD-L1 therapy. J. Immunother. Cancer.

[5] Feng Z., Bethmann D., Kappler M., Ballesteros-Merino C., Eckert A., Bell R.B., Cheng A., Bui T., Leidner R., Urba W.J. (2017). Multiparametric immune profiling in HPV– oral squamous cell cancer. JCI Insight.

[6] Patel S.S., Weirather J.L., Lipschitz M., Lako A., Chen P.H., Griffin G.K., Armand P., Shipp M.A., Rodig S.J. (2019). The microenvironmental niche in classic Hodgkin lymphoma is enriched for CTLA-4–positive T cells that are PD-1–negative. Blood.

[7] Topalian S.L., Bhatia S., Amin A., Kudchadkar R.R., Sharfman W.H., Lebbé C., Delord J.P., Dunn L.A., Shinohara M.M., Kulikauskas R. (2020). Neoadjuvant Nivolumab for Patients with Resectable Merkel Cell Carcinoma in the CheckMate 358 Trial. J. Clin. Oncol..

[8] Helmink B., Reddy S., Gao J., Zhang S., Basar R., Thakur R., Yizhak K., Sade-Feldman M., Blando J., Han G. (2020). B cells and tertiary lymphoid structures promote immunotherapy response. Nature.

[9] Berry S., Giraldo N., Green B., Cottrell T., Stein J., Engle E., Xu H., Ogurtsova A., Roberts C., Wang D. (2021). Analysis of multispectral imaging with the AstroPath platform informs efficacy of PD-1 blockade. Science.

[10] Tumeh P.C., Harview C.L., Yearley J.H., Shintaku I.P., Taylor E.J.M., Robert L., Chmielowski B., Spasic M., Henry G., Ciobanu V. (2014). PD-1 blockade induces responses by inhibiting adaptive immune resistance. Nature.

[11] Herbst R.S., Soria J.C., Kowanetz M., Fine G.D., Hamid O., Gordon M.S., Sosman J.A., McDermott D.F., Powderly J.D., Gettinger S.N. (2014). Predictive correlates of response to the anti-PD-L1 antibody MPDL3280A in cancer patients. Nature.

[12] Chen P.L., Roh W., Reuben A., Cooper Z.A., Spencer C.N., Prieto P.A., Miller J.P., Bassett R.L., Gopalakrishnan V., Wani K. (2016). Analysis of Immune Signatures in Longitudinal Tumor Samples Yields Insight into Biomarkers of Response and Mechanisms of Resistance to Immune Checkpoint Blockade. Cancer Discov..

[13] Forde P.M., Chaft J.E., Smith K.N., Anagnostou V., Cottrell T.R., Hellmann M.D., Zahurak M., Yang S.C., Jones D.R., Broderick S. (2018). Neoadjuvant PD-1 Blockade in Resectable Lung Cancer. N. Engl. J. Med..

[14] Lu S., Stein J.E., Rimm D.L., Wang D.W., Bell J.M., Johnson D.B., Sosman J.A., Schalper K.A., Anders R.A., Wang H. (2019). Comparison of Biomarker Modalities for Predicting Response to PD-1/PD-L1 Checkpoint Blockade: A Systematic Review and Meta-analysis. JAMA Oncol..

[15] Taube J.M., Roman K., Engle E.L., Wang C., Ballesteros-Merino C., Jensen S.M., McGuire J., Jiang M., Coltharp C., Remeniuk B. (2021). Multi-institutional TSA-amplified Multiplexed Immunofluorescence Reproducibility Evaluation (MITRE) Study. J. Immunother. Cancer.

[16] Taube J.M., Akturk G., Angelo M., Engle E.L., Gnjatic S., Greenbaum S., Greenwald N.F., Hedvat C.V., Hollmann T.J., Juco J. (2020). The Society for Immunotherapy of Cancer statement on best practices for multiplex immunohistochemistry (IHC) and immunofluorescence (IF) staining and validation. J. Immunother. Cancer.

[17] Deagle R.C., Wee T.L.E., Brown C.M. (2017). Reproducibility in light microscopy: Maintenance, standards and SOPs. Int. J. Biochem. Cell Biol..

[18] Montero Llopis P., Senft R.A., Ross-Elliott T.J., Stephansky R., Keeley D.P., Koshar P., Marqués G., Gao Y.S., Carlson B.R., Pengo T. (2021). Best practices and tools for reporting reproducible fluorescence microscopy methods. Nat. Methods.

[19] Sasaki A. (2022). Recent advances in the standardization of fluorescence microscopy for quantitative image analysis. Biophys. Rev..

[20] Akoya Biosciences inForm Product Note: Quantitative Pathology Imaging and Analysis, 2019. Software Product Note. https://www.akoyabio.com/wp-content/uploads/2021/12/akProdNote_InForm_v2.pdf.

[21] Bradski G. (2000). The OpenCV Library. Dr. Dobb’s Journal of Software Tools.

[22] Curti B.D., Faries M.B. (2021). Recent Advances in the Treatment of Melanoma. N. Engl. J. Med..

[23] Robert C., Ribas A., Schachter J., Arance A., Grob J.J., Mortier L., Daud A., Carlino M.S., McNeil C.M., Lotem M. (2019). Pembrolizumab versus ipilimumab in advanced melanoma (KEYNOTE-006): Post-hoc 5-year results from an open-label, multicentre, randomised, controlled, phase 3 study. Lancet Oncol..

[24] Larkin J., Chiarion-Sileni V., Gonzalez R., Grob J.J., Cowey C.L., Lao C.D., Schadendorf D., Dummer R., Smylie M., Rutkowski P. (2015). Combined Nivolumab and Ipilimumab or Monotherapy in Untreated Melanoma. N. Engl. J. Med..

[25] Wolchok J.D., Chiarion-Sileni V., Gonzalez R., Grob J.J., Rutkowski P., Lao C.D., Cowey C.L., Schadendorf D., Wagstaff J., Dummer R. (2022). Long-Term Outcomes with Nivolumab Plus Ipilimumab or Nivolumab Alone Versus Ipilimumab in Patients with Advanced Melanoma. J. Clin. Oncol..

[26] Food and Drug Administration (2023). List of Cleared or Approved Companion Diagnostic Devices (In Vitro and Imaging Tools). https://www.fda.gov/medical-devices/invitro-diagnostics/list-cleared-or-approved-companion-diagnostic-devices-invitro-and-imaging-tools.

[27] Hodi F.S., Chiarion-Sileni V., Gonzalez R., Grob J.J., Rutkowski P., Cowey C.L., Lao C.D., Schadendorf D., Wagstaff J., Dummer R. (2018). Nivolumab plus ipilimumab or nivolumab alone versus ipilimumab alone in advanced melanoma (CheckMate 067): 4-year outcomes of a multicentre, randomised, phase 3 trial. Lancet Oncol..

[28] Jørgensen J.T., Hersom M. (2018). Clinical and Regulatory Aspects of Companion Diagnostic Development in Oncology. Clin. Pharmacol. Ther..

[29] Locke D., Hoyt C.C. (2023). Companion diagnostic requirements for spatial biology using multiplex immunofluorescence and multispectral imaging. Front. Mol. Biosci..

[30] Food and Drug Administration (2014). In Vitro Companion Diagnostic Devices: Guidance for Industry and Food and Drug Agministration Staff. https://www.fda.gov/regulatory-information/search-fda-guidance-documents/invitro-companion-diagnostic-devices.

