# Supplementary material for: Comparing and Correcting Spectral Sensitivities between Multispectral Microscopes: A Prerequisite to Clinical Implementation
Source: Cancers (Basel). 2023 Jun 8;15(12):3109. doi: 10.3390/cancers15123109 (PMC10296646; doi:10.3390/cancers15123109)
Supplement: Supplementary file 1 [file cancers-15-03109-s001.zip › cancers-2394778-supplementary.pdf]

Table S1: Antibodies and staining conditions for automated mIF assay. Formalin-fixed, paraffin-embedded tissues melanoma samples were stained for a mIF 8-plex assay plus DAPI.

	Primary Antibody						Secondary Antibody			TSA Fluorophore		
**Position**	**Marker**	**Species**	**Clone**	**Source**	**Dilution** (μg/mL)	**Incubation Time (min)**	**Amplification**	**Source**	**Dilution (in 1x TBS)**	**Opal**	**TSA Dilution**	**Excitation Filter**
1	FoxP3	Mouse	236A/E7	abcam	100	60	Mouse Powervision	Leica Biosystems	1:3	540	300	FITC
2	CD8	Mouse	4B11	Leica Biosystems	200	30	Opal	Akoya Biosciences	None	570	150	Cy3
3	CD3	Mouse	LN10	Leica Biosystems	50	60	Opal	Akoya Biosciences	None	480	150	Opal 480
4	PD1	Rabbit	EPR4877(2)	abcam	1000	60	Rabbit Powervision	Leica Biosystems	1:3	650	250	Cy5
5	PDL1	Rabbit	SP142	abcam	50	60	Rabbit Powervision	Leica Biosystems	1:3	520	250	FITC
6	CD163	Mouse	EDHu-1	Leica Biosystems	800	60	Rabbit Powervision	Leica Biosystems	1:3	690	150	Cy5
7	Sox10<br>S100	Mouse<br>Mouse	BC34<br>4C4.9	BioCare Medical<br>Abnova	400<br>1600	30	Opal	Akoya Biosciences	None	620	150	Texas Red
8	ATPase<br>CD44<br>CD45	Rabbit<br>Mouse<br>Mouse	EP1845Y<br>DF1485<br>2B11+PD7/26	abcam<br>Dako<br>Dako	100<br>100<br>200	30	Rabbit/ Mouse Powervision	Leica Biosystems	1:1 (Mouse: Rabbit)	TSA-DIG<br>DIG-780<br>DAPI	100<br>25<br>12.5	Opal 780<br>DAPI

Table S2: Dates that each sample was scanned on the three microscopes.

**Sample**	**Microscope 1** Scan 1	Scan 2	**Microscope 2** Scan 1	Scan 2	**Microscope 3** Scan 1	Scan 2
Sample 1	Jan 6 2022	Jan 2 2022	Dec 6 2021	Dec 14 2021	Dec 22 2021	Dec 25 2021
Sample 2	Jan 4 2022	Dec 29 2021	Dec 7 2021	Dec 12 2021	Dec 22 2021	Dec 25 2021
Sample 3	Jan 5 2022	Jan 1 2022	Dec 4 2021	Dec 12 2021	Dec 20 2021	Dec 24 2021
Sample 4	Jan 7 2022	Dec 30 2021	Dec 3 2021	Dec 11 2021	Dec 19 2021	Dec 22 2021
Sample 5	Jan 5 2022	Jan 2 2022	Dec 5 2021	Dec 13 2021	Dec 21 2021	Dec 25 2021
Sample 6	Jan 6 2022	Jan 3 2022	Dec 4 2021	Dec 13 2021	Dec 20 2021	Dec 28 2021
Sample 7	Jan 5 2022	Jan 2 2022	Dec 5 2021	Dec 14 2021	Dec 21 2021	Dec 25 2021
Sample 8	Jan 7 2022	Jan 3 2022	Dec 5 2021	Dec 13 2021	Dec 21 2021	Dec 29 2021

Table S3: Number of HPFs generated for every scan of each sample used.

**Sample**	**Microscope 1** Scan 1	Scan 2	**Microscope 2** Scan 1	Scan 2	**Microscope 3** Scan 1	Scan 2
Sample 1	563	578	586	592	595	577
Sample 2	553	579	563	577	576	567
Sample 3	892	885	905	898	892	900
Sample 4	659	662	680	669	680	667
Sample 5	652	643	646	649	636	616
Sample 6	705	698	721	722	714	700
Sample 7	1010	1018	1022	1016	1006	999
Sample 8	706	711	714	717	708	701
Total	5740	5774	5837	5840	5807	5727

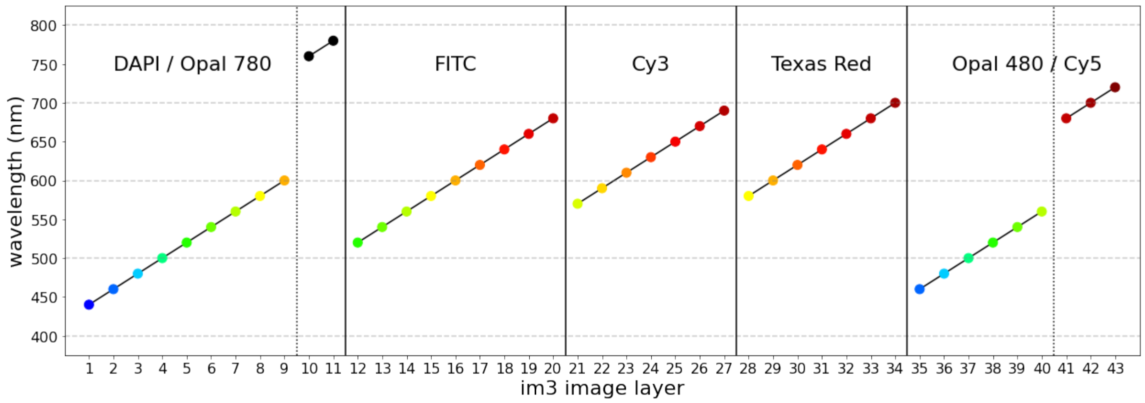

Figure S1: The narrow-band filter wavelengths contributing to each HPF image layer. Data points are colored by their wavelength in the visible spectrum where applicable. Divisions are shown between groups of layers imaged using different broadband filters.

A

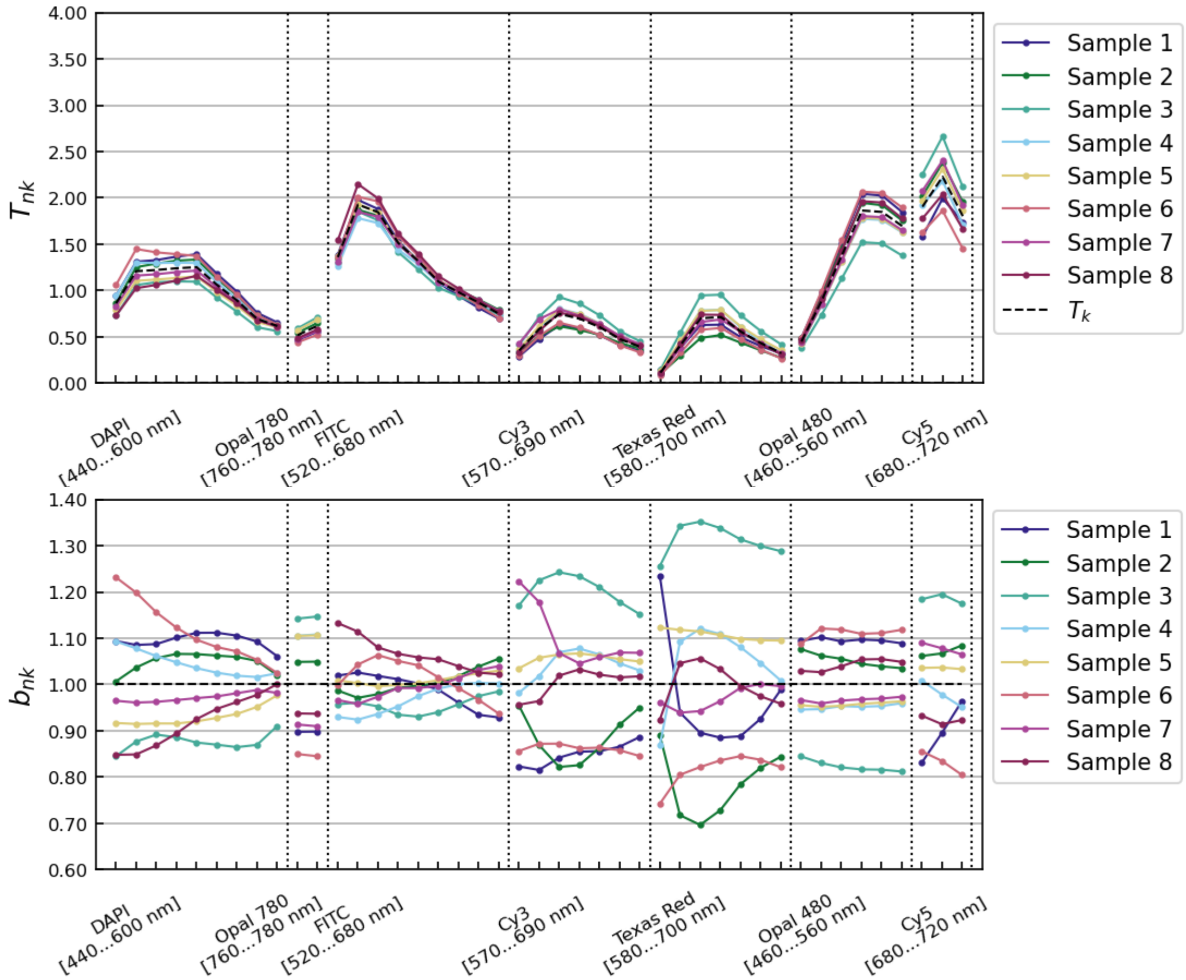

Figure S2: (**A**) The T_{nk} spectra representing relative variations about the mean (T_k) of the set of tissue samples. (**B**) The b_{nk} correction factors.

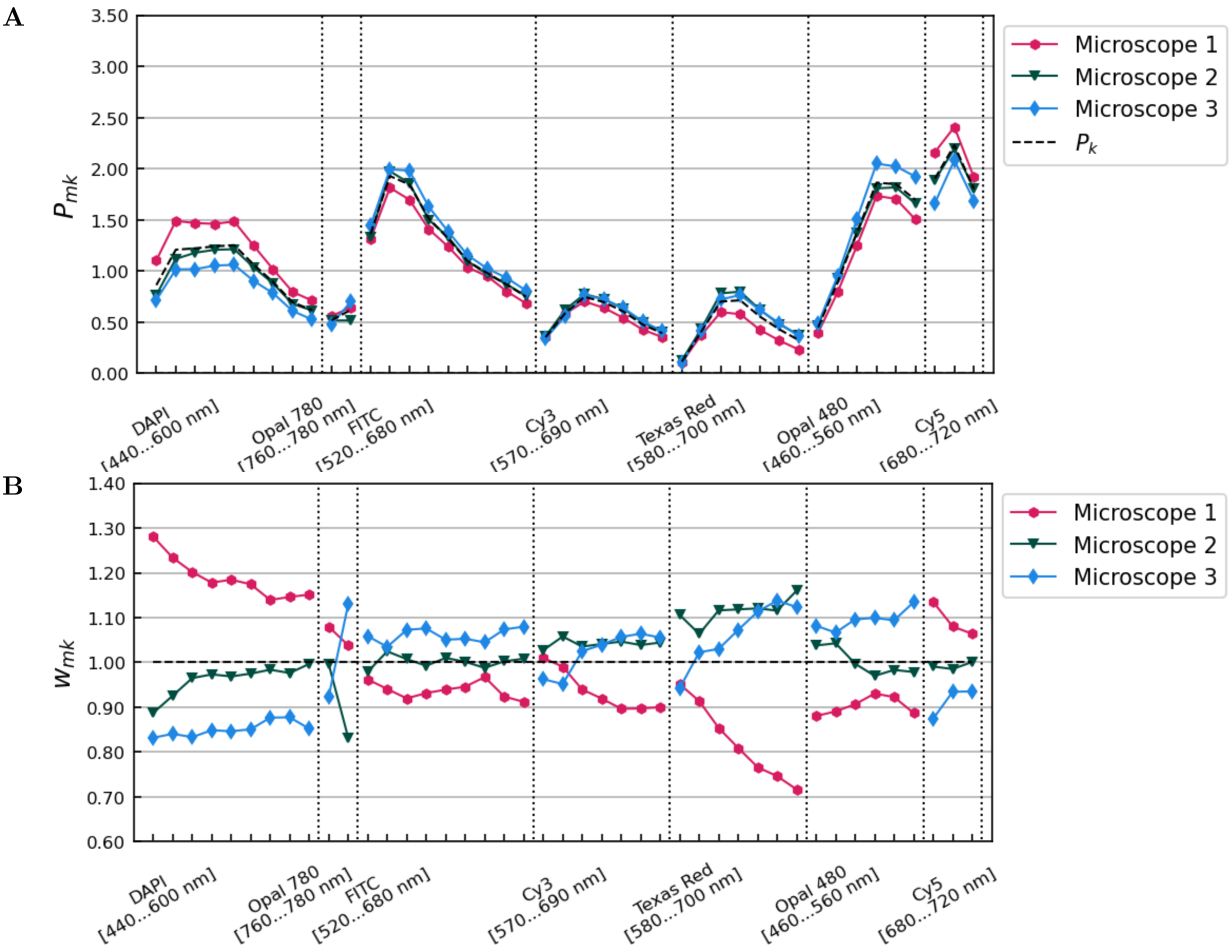

Figure S3: (**A**) The P_{mk} spectra representing relative variations about the mean (P_k) of all average microscope fluxes. (**B**) The w_{mk} correction factors.

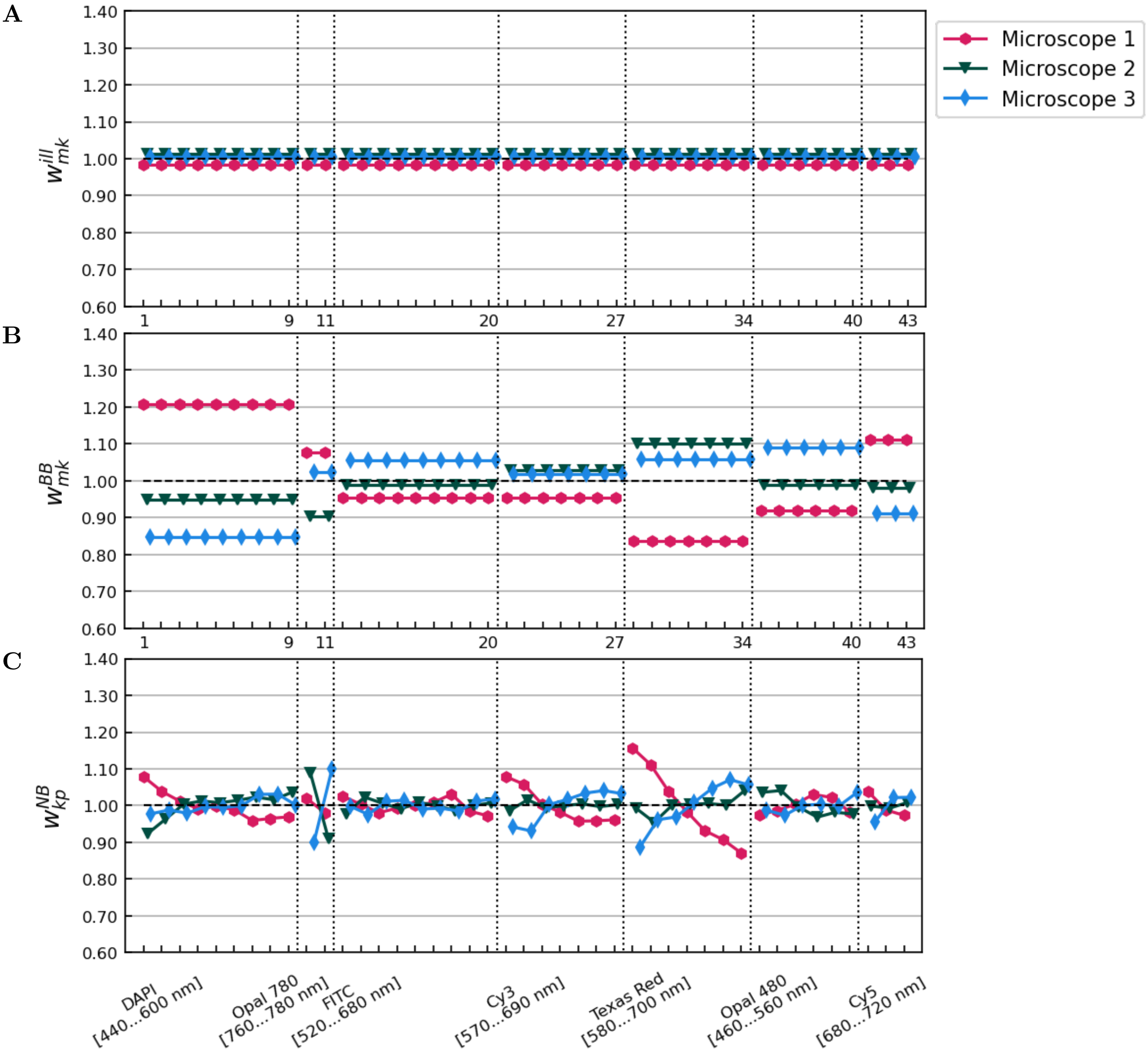

Figure S4: The factorized w_m^{ill} (**A**), w_{mk}^{BB} (**B**), and w_{mk}^{NB} (**C**) contributions to the total w_{mk} correction factors, showing that most of the wavelength-dependent differences between microscopes are limited to inhomogeneities between the broadband filters installed in each microscope.

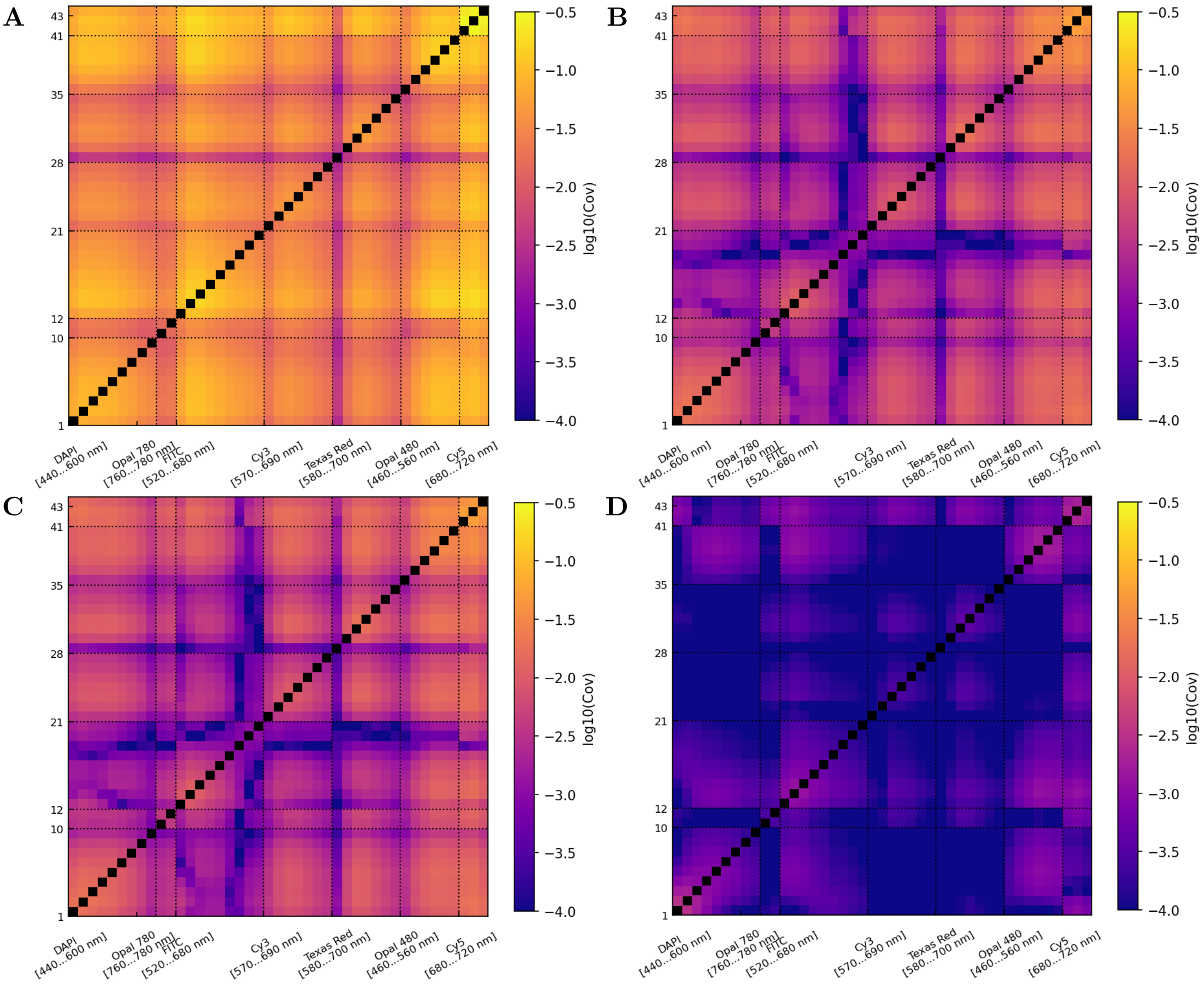

Figure S5: The image layer-projected covariance matrices at each stage of correction: (**A**) $\Sigma_{k_1k_2}(X)$ (normalization only), (**B**) $\Sigma_{k_1k_2}(x)$ (after amplitude correction), (**C**) $\Sigma_{k_1k_2}(y)$ (after correction with tissue profiles), and (**D**) $\Sigma_{k_1k_2}(z)$ (after correction with microscope profiles).

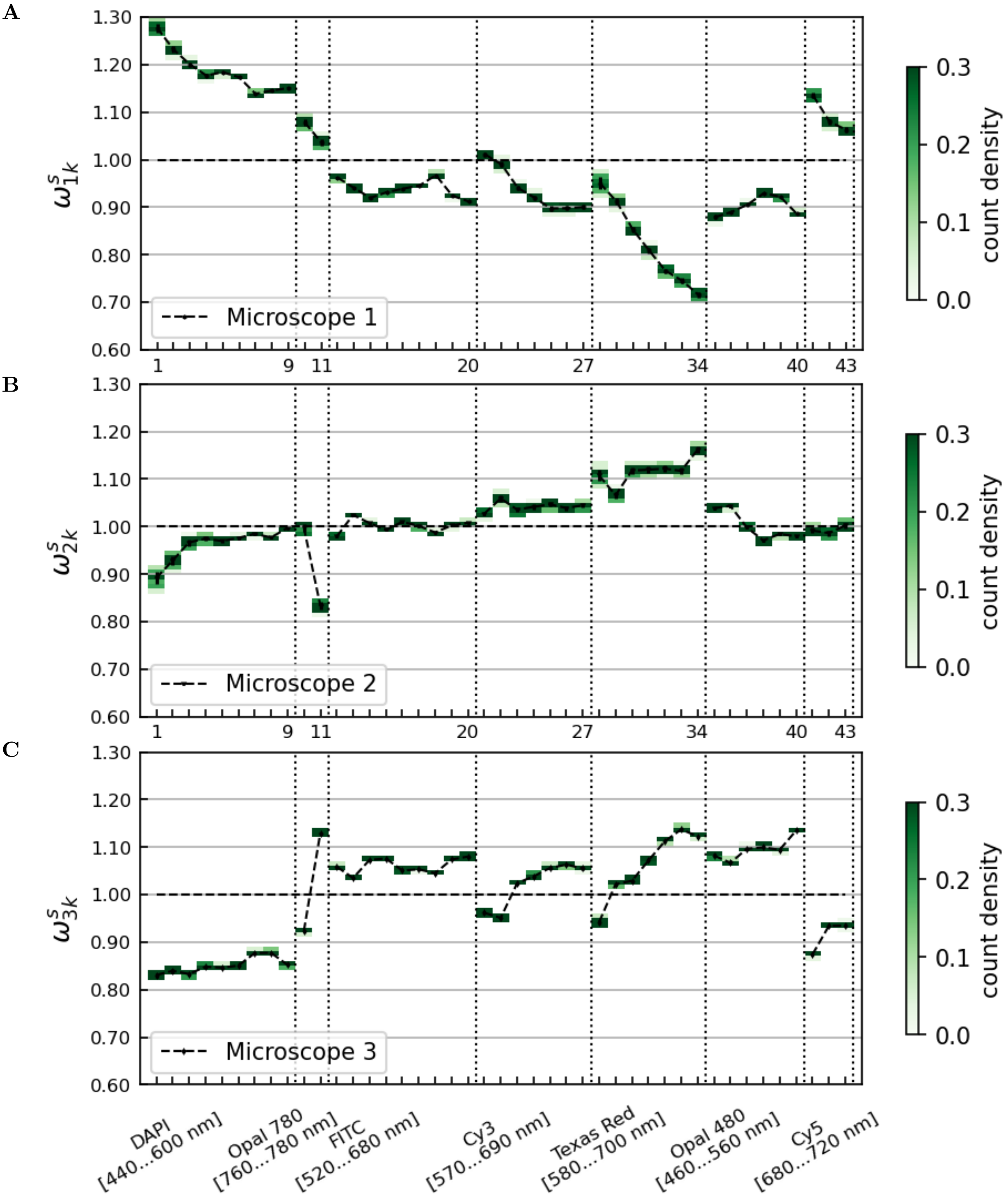

Figure S6: The microscope-dependent $C_m^s \omega_{mk}^s$ correction factors found in each iteration of the bootstrapping procedure for microscopes 1 (**A**), 2 (**B**), and 3 (**C**). The means of the distributions found are plotted as data points with standard deviation-sized error bars on top of a density histogram showing the spread of all values.

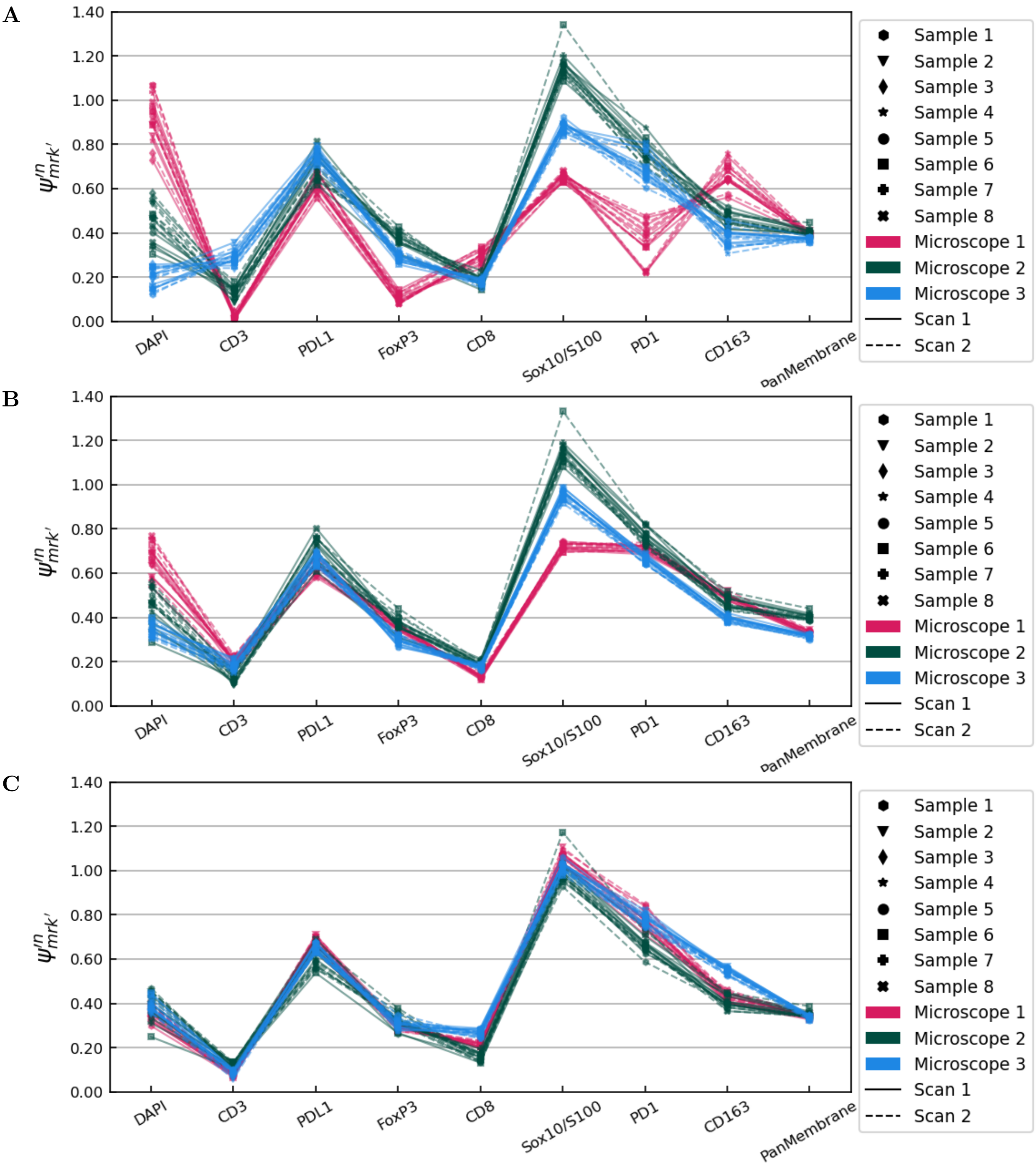

Figure S7: (**A**) The $\psi'^n_{mrk'}$ spectra obtained by unmixing raw images using a single library from microscope 2. (**B**) The $\psi'^n_{mrk'}$ spectra obtained by unmixing raw images using libraries corresponding to each imaging microscope. (**C**) The $\psi'^n_{mrk'}$ spectra obtained by applying factors to standardize data from multiple microscopes to the microscope 2 reference, and unmixing the standardized images using the microscope 2 library. Data from different microscopes are plotted in different colors. Solid and dashed lines show data from scans 1 and 2, respectively. Individual tissue samples are distinguished using different marker styles, as shown in the legend.
